# Novel structural co-expression analysis linking the *NPM1*-associated ribosomal biogenesis network to chronic myelogenous leukemia

**DOI:** 10.1038/srep10973

**Published:** 2015-07-24

**Authors:** Lawrence WC Chan, Xihong Lin, Godwin Yung, Thomas Lui, Ya Ming Chiu, Fengfeng Wang, Nancy BY Tsui, William CS Cho, SP Yip, Parco M. Siu, SC Cesar Wong, Benjamin YM Yung

**Affiliations:** 1 Department of Health Technology and Informatics, The Hong Kong Polytechnic University, Hong Kong; 2 Department of Biostatistics, School of Public Health, Harvard University, Massachusetts, USA; 3 Department of Clinical Oncology, Queen Elizabeth Hospital, Hong Kong

## Abstract

Co-expression analysis reveals useful dysregulation patterns of gene cooperativeness for understanding cancer biology and identifying new targets for treatment. We developed a structural strategy to identify co-expressed gene networks that are important for chronic myelogenous leukemia (CML). This strategy compared the distributions of expressional correlations between CML and normal states, and it identified a data-driven threshold to classify strongly co-expressed networks that had the best coherence with CML. Using this strategy, we found a transcriptome-wide reduction of co-expression connectivity in CML, reflecting potentially loosened molecular regulation. Conversely, when we focused on *nucleophosmin 1* (*NPM1*) associated networks, *NPM1* established more co-expression linkages with BCR-ABL pathways and ribosomal protein networks in CML than normal. This finding implicates a new role of *NPM1* in conveying tumorigenic signals from the BCR-ABL oncoprotein to ribosome biogenesis, affecting cellular growth. Transcription factors may be regulators of the differential co-expression patterns between CML and normal.

Gene co-expression networks can be used to investigate the inter-gene associations in expression profiles, reflecting functional linkages and potential coordinate regulations. Studies in recent years have proposed pairwise and structural analysis of co-expression^1^,^2^,^3^,^4^,^5^,^6^,^7^,^8^,^9^. The majority of these studies identify differential co-expression patterns between disease and healthy states based on the correlation coefficients among genes^4^. For pairwise analysis, two genes are linked if their correlation exceeds a specific threshold. To date, the existing approaches for optimizing the threshold aim to control the false discovery rate (FDR) or minimize the network complexity^1^,^5^. An optimal coherence of co-expression patterns with disease has not been achieved.

The co-expression structure is defined as the distribution of co-expression levels for a group of genes over a state. Structural analysis seeks to identify a group of genes whose co-expression structure in one state (e.g., neoplastic subjects) is significantly different from that in another state (e.g., normal subjects)^8^. For instance, gene set co-expression analysis (GSCA) was introduced to test for differential co-expression patterns between two states in a gene set based on gene ontology (GO) or a pathway using a dispersion index^8^. Significant differential co-expression patterns were identified by estimating the FDR after evaluating the exhaustive permutations of the samples^8^. Such an approach can indicate whether the observed differential co-expression patterns in a set of genes are obtained by chance. However, the approach does not provide information about which individual gene pairs in the set are strongly or weakly co-expressed and which network connections are altered because of the disease.

Here, we propose a statistical and graphical strategy for analyzing and classifying all individual gene pairs in a set of genes based on the differences between the co-expression structures of neoplastic and normal states (Fig. 1). For validation, we consider chronic myelogenous leukemia (CML) as a paradigm for targeted therapy and analyze a publicly available gene expression data of bone marrow mononuclear cells that have been collected from nine newly diagnosed CML patients and eight healthy volunteers. Briefly, CML is characterized by the Philadelphia (Ph) chromosome, which results from t(9;22)(q34;q11) balanced reciprocal translocation and leads to the formation of the *BCR-ABL* oncogene. The signaling pathways activated by *BCR-ABL* include the mitogen-activated protein kinase (MAPK) pathway, Janus-activated kinase (JAK)–STAT pathway and phosphoinositide 3-kinase (PI3K)/AKT pathway. All three activations lead to aberrant protein synthesis and deregulated cell growth^10^. Although conventional tyrosine kinase inhibitors (TKI) that target the TK activity of BCR-ABL oncoprotein are the first choice of treatment for CML, the drug responses are generally short-lived, and drug resistance remains a significant clinical problem. Hence, our understanding of CML is still rudimentary, and a better understanding of various signaling pathways involved in its pathogenesis may encourage the discovery of potential targets for a more effective treatment strategy. Our proposed method enhances the existing approach of structural co-expression analysis by identifying potential drug targets whose cooperativities on the BCR-ABL pathway are potent.

Nucleophosmin 1 (NPM1), also known as nucleolar phosphoprotein B23, is an important protein in the nucleophosmin/nucleoplasmin family of nuclear chaperones because NPM1 has deregulated expression in solid tumors and mutation or translocation in hematological malignancies^11^. NPM1 is also a versatile protein that participates in numerous cellular processes critical to cell growth and proliferation, including ribosomal RNA (rRNA) processing, ribosome biogenesis, and nuclear export of ribosomal subunits^12^,^13^. As a mitogen-induced protein, it responds to signals from the MAPK and PI3K/AKT pathways that are initiated by oncogenic Ras, promoting ribosome biogenesis and protein translation. This evidence suggests that NPM1 is strongly associated with the MAPK and PI3K/AKT pathways for ribosome biogenesis, and it may play a critical role in 1) monitoring the stress experienced by the cell and 2) modulating the molecular mechanisms related to cell growth, proliferation and survival. To test this hypothesis, we applied the proposed method to quantify and compare the state-specific associations of *NPM1* gene expression with gene expressions from the combined BCR-ABL/MAPK/PI3K/AKT set of pathways. To further explore the role of *NPM1* in ribosome biogenesis, we analyzed the co-expression network of 93 *NPM1*-associated genes that were defined in the Molecular Signature Database (MSigDB) as a gene cluster covering most of the ribosomal proteins^14^. Cell line experiments were performed to validate the strong co-expressions with *NPM1*, termed *NPM1*-doublets. Using the Prediction of Transcriptional Regulatory Modules (PReMod) database^15^, we identified transcription factors (TFs) that concurrently target the *NPM1*-doublets and elucidated their effect on co-expression patterns. Finally, we performed functional annotation analysis to decipher the underlying *NPM1*-associated mechanism in CML.

## Results

### Global co-expression structure of CML

We studied the co-expression structure of CML using a microarray dataset from Diaz-Blaco *et al.* (GEO accession number GSE5550)^16^. The dataset consisted of a Caucasian cohort of nine untreated Ph+ CML patients and eight healthy controls. Total RNA extracted from CD34+ bone marrow mononuclear cells was analyzed by Affymetrix HG-Focus GeneChips, which interrogated 8,537 well-characterized human genes. The raw expression intensities were normalized using variance stabilizing transformation (VST), an algorithm supported by the affy package of ‘R’ functions integrated into Bioconductor^16^,^17^.

We constructed the transcriptome-wide co-expression structure of CML using expression data from the CML patients. The structure consists of Pearson correlation coefficients (r) of all possible unique pair combinations of the 8,537 genes. This resulted in a profile of the r values of 36,435,916 gene pairs (doublets).

We first investigated whether CML patients had a co-expression structure that was different from healthy individuals. Hence, we constructed another co-expression structure using expression data from the healthy controls. A significant difference in the empirical distributions of |r| was observed between the CML and normal co-expression structures (two-sample Kolmogorov-Smirnov test, D ≫ D_0.05_, i.e., *P* < 0.05 where D_0.05_ is the empirical threshold). The result suggests that there was a global disturbance of the co-expression connections in CML.

We then sought to classify the doublets into those that were strongly or weakly co-expressed. Conventionally, a fixed *P*-value cutoff was used to define the presence or absence of co-expression between the two gene members of a doublet. However, such a method statistically controls the false co-expression discovery of individual doublets only, but it ignores the quantitative measure of the coherence of the doublets to either disease or normal. Here, we used a data-driven approach to determine a dataset-specific threshold of the r value for classifying strongly or weakly co-expressed doublets that were relevant to the CML and normal samples in the dataset. As shown in Supplementary Fig. 1, the cumulative distributions of |r| were maximally different between CML and normal at a threshold (Ĉ) of 0.400. Using this threshold, a total of 12 million and 23 million strongly co-expressed doublets were identified in CML and normal, respectively (Supplementary Table 1). As the prevalence of strongly co-expressed doublets was significantly reduced in CML (log (OR) = −0.566, *P* < 0.001), we suggested that CML might be related to a transcriptome-wide breakdown of co-expression regulation.

A co-expression galaxy was formed by sketching the scatter plot of the r values of the normal state against CML. By partitioning the co-expression galaxy with the threshold, we identified two important sets of co-expressed doublets that had strong co-expression in CML but not normal (CML-specific doublets) and vice versa (normal-specific doublets) (Supplementary Fig. 2). These doublets are potentially relevant to the disease, and they would be of biological and clinical value.

### Over co-expression of *NPM1* with BCR-ABL relevant pathways

To further determine the biological implication of the doublets identified by global analysis, we examined the doublets formed between *NPM1* and gene members of the MAPK and PI3K/AKT pathways, which are relevant to the oncogenic BCR-ABL fusion protein. We found that *NPM1* had established ten CML-specific doublets, and there were only two normal-specific doublets, with the pathways (Fig. 2). Based on this observation, we speculated that BCR-ABL and its relevant pathways might be falsely over-connected with *NPM1*. Additional cellular growth and proliferation pathways may in turn be activated through the mediation of *NPM1* in CML.

### CML-specific co-expression of *NPM1* with the ribosomal protein network

The over co-expression relationship between *NPM1* and the BCR-ABL pathways prompted us to systematically investigate the *NPM1*-focused co-expression structure. Ninety-three genes in the neighborhood of *NPM1* were selected from the Molecular Signature Database (GCM_NPM1 gene set)^14^. CML and normal co-expression structures were constructed as described above using these 93 genes, including *NPM1*, resulting in r values of 4,278 doublets for each of the structures (Supplementary Fig. 3). The two co-expression structures were significantly different from one another (two-sample Kolmogorov-Smirnov test, D ≫ D_0.05_, i.e., *P *< 0.05), with a data-driven threshold of Ĉ = 0.252. With reference to this threshold, the prevalence of strongly co-expressed doublets was significantly increased in CML compared to normal (log (OR) = 0.227, *P *< 0.001) (Supplementary Table 2). It is worth noting that this trend of a CML-associated increase of *NPM1* co-expression is the opposite of that found in the transcriptome-wide co-expression structure in which a general reduction of connectivity was observed in CML (Supplementary Table 1). The finding indicates that *NPM1* may mediate various false connections of the originally discrete networks, which may be oncogenic if they are synergistically activated in CML.

In total, we identified 11 normal- and 69 CML-specific doublets from the co-expression structures, which include 6 and 21 *NPM1*-doublets respectively (Fig. 3). All of the 21 CML-specific doublets were validated by real-time quantitative PCR with the use of the K562 CML cell line. Upon resveratrol treatment, the level of *NPM1* mRNA was significantly decreased compared with those treated with the vehicle control (DMSO) (t-test, *P* < 0.05) (Supplementary Fig. 4). Notably, significant reductions were also observed in the expression levels of the 21 mRNAs that were co-expressed with *NPM1* (t-test, *P* < 0.05 for all genes) (Supplementary Fig. 4). According to the co-expression structure analysis, these 21 mRNAs were all positively correlated with *NPM1* in CML (Fig. 3b). The same trend of resveratrol-repression for *NPM1* and its co-expressed mRNAs confirmed the structural co-expression finding shown in Fig. 3.

We inspected the biological function of the normal- and CML-specific *NPM1*-doublets (Fig. 3) and found that three RNAs coding for ribosomal protein (RP), i.e., *ribosomal protein L10a* (*RPL10A)*, *ribosomal protein L31* (*RPL31)* and *ribosomal protein L36a* (*RPL36A)*, were only present in CML-specific doublets and were not present in normal-specific doublets. This observation is interesting because NPM1 protein is a well-recognized key player in ribosome biogenesis and transport^11^. The whole *NPM1*-focused co-expression structure involved a total of 33 RP genes. We further retrieved the co-expression information of these RP genes and found that *RPL10A*, *RPL31* and *RPL36A* were co-expressed with a relatively large network of 23 RP mRNAs (Fig. 3b). Meanwhile, for normal-specific doublets, there was only a small network of 6 RP genes, and none of them were co-expressed with *NPM1* (Fig. 3a). Our finding suggests that a co-expression network of RP genes may be established during CML development, and the network may further connect to *NPM1* through the hubs of *RPL10A*, *RPL31* and *RPL36A.* The aforementioned CML-specific doublets were statistically examined using the one-sample t-test. All the discovered connections were found to be reliable (FDR ≤ 0.07, Supplementary Table 3). As this work focuses on exploring the synergistic perturbation of the structural co-expression profile for CML, the paired t-test was performed, indicating a significant difference in the Fisher-transformed r between the CML and normal states over all of these CML-specific doublets (t = 17.52, p = 6.49 × 10^−27^, Supplementary Table 4). These findings imply that the connections between NPM1 and RP genes are synergistically promoted in CML states compared with normal states.

We mapped 25 of the 26 CML-specific RP genes (Fig. 3b) onto the KEGG “Ribosome” network of MSigDB^14^. Notably, the *NPM1*-coexpressed *RPL10A, RPL31* and *RPL36A* were the 1^st^, 3^rd^ and 4^th^ top hub genes of the KEGG network (Supplementary Fig. 5). This finding further illustrates the controlling role of *RPL10A, RPL31* and *RPL36A* in ribosome biogenesis. Their co-expression with *NPM1* possibly transfers the oncogenic signal from the BCR-ABL pathways (Fig. 2) to aberrant ribosome biogenesis, affecting protein synthesis and cell growth in CML.

In addition to the ribosome, the normal- and CML-specific *NPM1*-doublets were in fact associated with a total of 20, 25 and 2 functional annotations of the GeneSetDB (GO)^18^,^19^, Reactome pathway database^20^, and MSigDB (KEGG)^14^, respectively (Fisher’s exact test, Bonferroni adjusted *P* < 0.05) (Supplementary Tables 5-7). Their association with CML would also be worth exploring in the future.

### Transcription factors as regulators of co-expression

One of the biological mechanisms that coordinate gene co-expression operates through TFs. Hence, for each strongly co-expressed *NPM1*-doublet (Fig. 3), we predicted the responsible TFs from the PReMod database^15^ (Supplementary Table 8). We found that the predicted TFs that regulate of the normal- and CML-specific doublets largely overlapped. The common TFs include cyclic AMP-responsive element-binding protein 1 (CREB1), E2F transcription factor 1 (E2F1), E2F transcription factor 3 (E2F3), E2F transcription factor 4 (E2F4), nucleosome-remodeling factor subunit BPTF (FALZ), protein MAX (MAX), myc proto-oncogene protein (MYC), paired box protein (PAX2), signal transducer and activator of transcription 5A (STAT5A), transcription factor Dp-1 (TFDP1) and zinc finger E-box-binding homeobox 1 (ZEB1) (Fig. 4). These 11 TFs collectively controlled 50% and 52% of the normal- and CML-specific *NPM1* doublets, respectively. Importantly, with the shared TFs, the direction of co-expression was reversed for the CML- and normal-specific *NPM1*-doublets. For CML, all of the 13 doublets that were the predicted targets of the TFs were positively co-expressed with *NPM1*, while for normal, three of the four doublets (75%) were negatively co-expressed with *NPM1* (Fig. 4). This finding suggests that the same set of TFs may exert opposite effects of co-expression in normal versus CML states^21^.

## Discussion

We introduced a structural approach to graphically compare the transcriptome-wide co-expression patterns between CML and normal states as well as to determine a state-coherent threshold for identifying doublets that were alternatively co-expressed in CML.

The transcriptome-wide analysis revealed a general reduction in the co-expressed doublets in CML, suggesting a possible loosening of the network regulation in cancer. On the other hand, the *NPM1*-associated co-expression network was enlarged in CML. Because NPM1 protein is an early sensor of oncogenic stress^11^, *NPM1* possibly has a cooperative role in joining and activating multiple tumorigenic pathways via co-expression. In particular, when we focused on *NPM1*-doublets that were uniquely lost or invoked in CML, we found that *NPM1* was exceedingly co-expressed with the mRNAs of BCR-ABL related pathways and ribosomal hub proteins (*RPL10A*, *RPL31* and *RPL36A*). Hence, *NPM1* may be an important mediator, connecting the BCR-ABL network to ribosome biogenesis and, hence, protein synthesis and cell growth.

We used resveratrol as an external stress on K562 CML cell lines to investigate the 21 CML-specific *NPM1*-doublets identified by the co-expression analysis (Fig. 3b). Resveratrol has been reported as a potent growth inhibitor in various human cell lines^22^. It represses mTOR, which is a downstream component of the BCR-ABL associated MAPK and PI3K/AKT pathways, and inhibits global protein synthesis^22^. We demonstrated here that upon resveratrol treatment, down-regulated expression was found for *NPM1* and all of its 21 co-expressed mRNAs, including those encoding ribosomal hub proteins (*RPL10A*, *RPL31* and *RPL36A*). This finding provides insight into the mechanism of BCR-ABL-associated cell growth that *NPM1* may be a regulator downstream of mTOR. In pharmaceutical development, the search of downstream targets of BCR-ABL that are essential for cell proliferation and survival is important in drug design^23^. After clarifying the pathogenic mechanism, *NPM1* is a conceivable molecular target for CML treatment.

In addition to mRNAs of ribosomal proteins, we also identified the co-expression association of *NPM1* with transcripts of other functions (Fig. 3b). One of them is the mRNA of heterogeneous nuclear ribonucleoprotein *hnRNPM*. Dery *et al.* reported that hnRNPM, together with hnRNPA1 and huRNPL, controls the alternative splicing of pre-mRNA of *carcinoembryonic antigen related cell adhesion molecule 1* (CEACAM1), which is aberrantly expressed during carcinogenesis^24^. The co-expression of *NPM1* and *hnRNPM* is a novel observation because NPM1 has only previously been reported to interact with hnRNPU and hnRNPA1 in mRNA processing^12^. Our findings implicate another connection of BCR-ABL to hnRNP control and, hence, splicing through *NPM1* co-expression. Maggi *et al.* reported that the NPM1 complex formed with RPs and hnRNPs might be involved in the nuclear export of 40S and 60S ribosomal subunits^25^.

Eleven TFs concurrently targeting both normal- and CML-specific networks of *NPM1*-doubles were identified. The dysregulation of these TFs may be a driver of the co-expression alternation in CML. Among these TFs, the E2F family members of E2F1, E2F3 and E2F4 targeted the largest number of the *NPM1*-doublets (Fig. 4). Therefore, it is valuable to further investigate their role in CML. In addition, the regulation cascade of the 11 identified TFs would also be worthwhile to elucidate.

In summary, this study demonstrates a novel structural co-expression network analysis platform, which allows for the establishment of a cooperativity model for exploring cancer pathogenesis and its potential *NPM1*-oriented treatment exploration (Fig. 5). The platform can readily be applied to other diseases for diagnostic, prognostic and therapeutic investigation.

## Methods

### Study design overview

**Figure 1:**
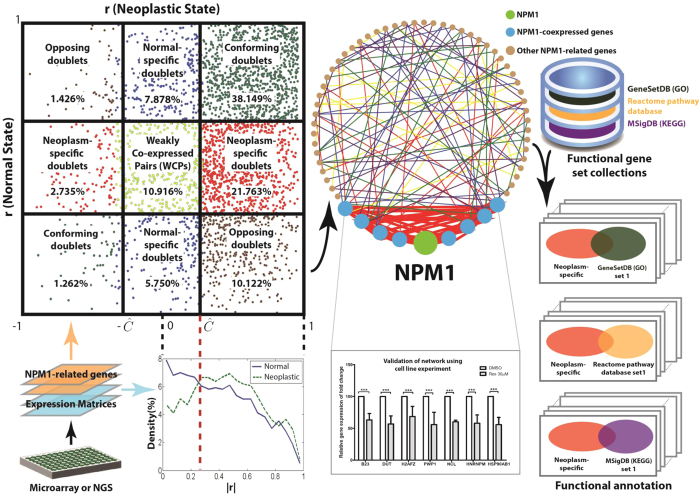
Overview of the proposed co-expression structural analysis strategy, experimental validation and functional annotation analysis. The colours of the points in the co-expression galaxy correspond to those of the lines in the co-expression networks. Red and blue colours represent neoplasm-specific and normal-specific doublets respectively. The red ellipse in functional annotation embraces a set of neoplasm-specific doublets as its items.

**Figure 2:**
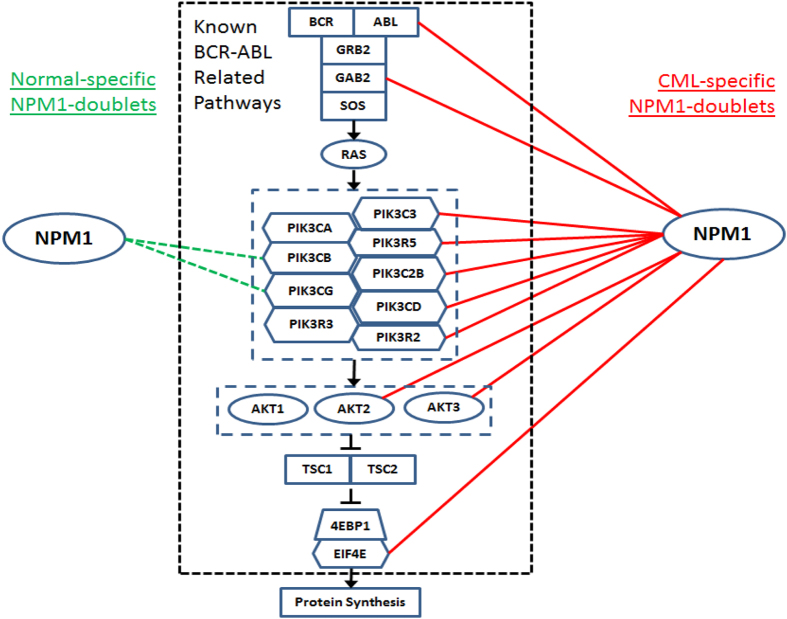
BCR-ABL related MAPK and PI3K/AKT pathways and their co-expression with NPM1. CML-specific and normal-specific *NPM1*-doublets are represented by red solid lines on the right and green dashed lines on the left respectively.

**Figure 3:**
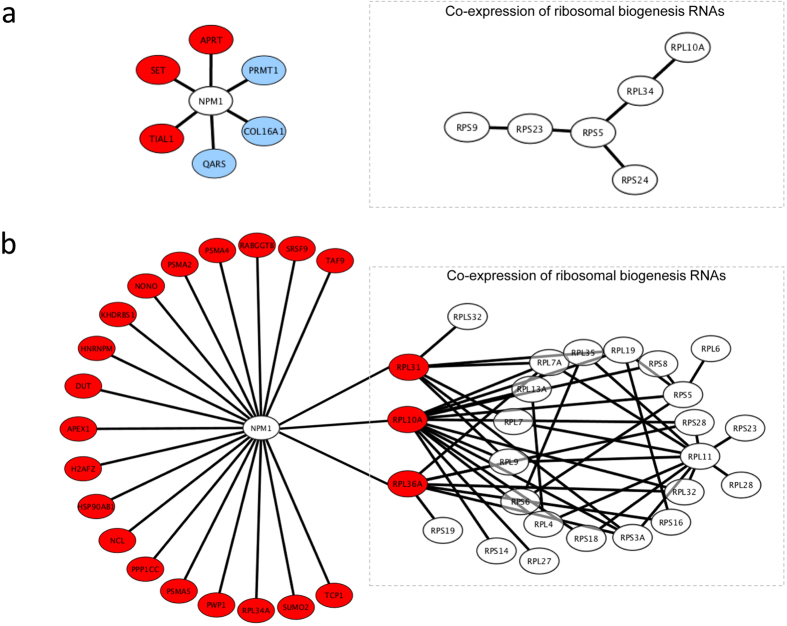
Co-expression networks of *NPM1*-doublets and RPs that were specifically found in (**a**) normal and (**b**) CML co-expression structures. Red circles represent RNAs positively correlated with *NPM1*, and blue circles represent RNAs negatively correlated with *NPM1*. RP co-expression networks are shown in dashed boxes. *RPL31*, *RPL10A* and *RPL36A* were hubs that connected RP network to *NPM1* in CML.

**Figure 4:**
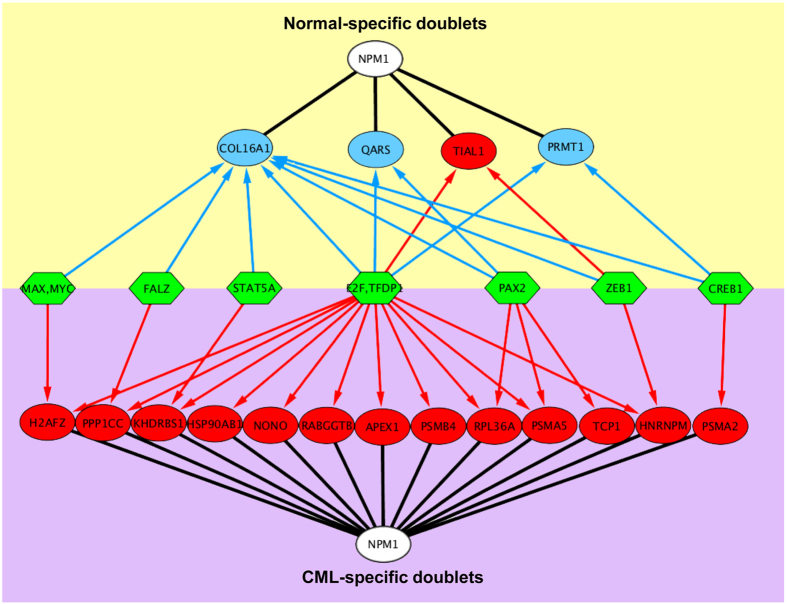
TFs concurrently targeted CML- and normal-specific *NPM1*-doublets. Green hexagons represent TFs. Red arrows represent the targeting of TFs to *NPM1*-doublets that were positively correlated (red circles), while blue arrows represent the targeting of TFs to *NPM1*-doublets that were negatively correlated (blue circles). E2F refers to E2F family members that included E2F1, E2F3 and E2F4.

**Figure 5:**
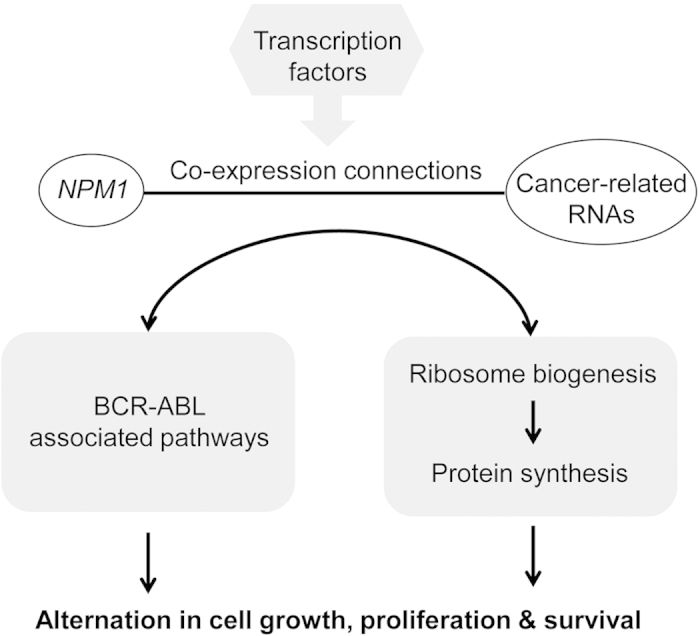
Proposed TF-driven cooperativity of *NPM1*-doublets in connection of BCR-ABL oncogenic signals to growth related activities in CML.

We defined and validated a strategy for (1) structural co-expression analysis, (2) doublet classification and (3) network analysis of the doublets that is based on the gene expression data collected from subjects in neoplastic and normal states. CML was considered the neoplasm of interest, and the strategy was applied to analyze a microarray dataset on the genomic scale and for the *NPM1*-related gene set. Among the networks identified with respect to various characteristics, the CML-specific network infers the mechanism of the disease and treatment response. Therefore, the real time PCR experiment on the CML cell line with resveratrol treatment was performed to further validate the CML-specific network. To decipher the underlying *NPM1*-oriented mechanism of disease and treatment in CML, the functional annotation analysis was performed on the identified network connections (or gene pairs) using the pathway/GO sets. Figure 1 illustrates the overview of the proposed strategy, experimental validation and functional annotation analysis. The TFs that concurrently target the *NPM1*-doublets were identified and their cooperative effects on *NPM1*-related co-expression were compared between the normal and CML groups.

### Expression and co-expression measures

The proposed strategy is applicable to the expression matrices derived from RNA-Seq or the microarray dataset. For RNA-Seq data, the expression of a gene is quantified by “reads per kilobase of exon model per million mapped reads” (RPKM), which normalizes the read measurement by the RNA length and total read number to ensure a fair comparison across samples^26^. For microarray data, the raw expression intensities are normalized using VST across the samples to ensure normality of the data and that the up and down regulations are equally treated^17^. Because the expression level of a gene is measured using one or multiple probes, the average intensity value is collected to further summarize and represent the expression level for each gene. Therefore, letting x_*ij*_ denote the expression level of the *i*^th^ gene and *j*^th^ sample of a state, an M×N expression matrix is formed for each state, where M is the number of genes, N is the number of samples of the same state, and each row in the matrix represents the expression profile of a gene across all N samples:
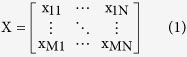


Assuming the expression intensities are normally distributed, the Pearson correlation coefficient *r*_*ij*_ measuring the co-expression between genes *i* and *j* is written as follows:
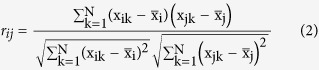
where 
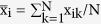
 and 
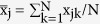
 are the mean expression levels of genes *i* and *j*, respectively. Given M genes, there are M×(M-1)/2 unique pairs of genes and correlation coefficients can be calculated.

### Structural co-expression analysis

Our classification of gene pairs into strongly or weakly co-expressed relies on the structural comparison of the distributions of co-expression levels or co-expression structures. The magnitudes of co-expressions are calculated by taking the absolute values of the Pearson correlation coefficients. That is, *C*(*i,j*) = | *r*_*ij*_ |. The co-expression level is denoted by *C*_*d*_(*i,j*) if the expression profiles of the *i*^th^ and *j*^th^ genes are extracted from the neoplastic state samples and *C*_*n*_(*i,j*) if the profiles are extracted from the normal state samples. To determine a co-expression threshold associated with the states, the approach implicitly tests the research hypothesis that the gene co-expression patterns of the neoplastic and normal states come from two different distributions. This hypothesis test uses structural analysis to determine whether the gene pairs in a state are more likely to exhibit a stronger co-expression structure than that in the other state. The two-sample Kolmogorov-Smirnov (KS) test was applied to examine the structural difference because it is sensitive to the deviation between the co-expression distribution profiles over a set of genes rather than that between individual gene pairs. Superior to other non-parametric tests, the two-sample KS test yields a threshold value at which the deviation between the cumulative distribution functions of *C*_*d*_ and *C*_*n*_ is maximal. More specifically, if we let *F*_*d*_, *F*_*n*_ and *D* denote the cumulative distribution functions (CDF) of *C*_*d*_ and *C*_*n*_ and the maximum deviation, respectively, *D* is given by:
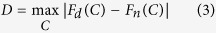


Note that the inequalities considered in the CDFs are reversed because our interest focuses on the strong co-expression.
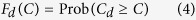

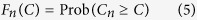


The optimal threshold, Ĉ, represents a co-expression magnitude at which *F*_*d*_ and *F*_*n*_ are extremely deviated. In a two-sample KS test, the test statistic *D* follows a chi-square distribution under the null hypothesis of no difference between the two cumulative distribution functions; therefore, the statistical significance can be tested by either comparing the calculated *p*-value with the desired alpha-level α or comparing *D* to a critical value *D*_α_^27^,
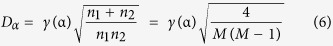
where *n*_1_ and *n*_2_ both equal to M × (M-1)/2, the number of gene pairs in neoplastic and normal states, and γ(α) is a function of α. According to Pearson and Hartley (1972)^27^, the value of γ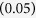 is 1.36. However, this value is appropriate when assuming that observations within each group are independent. Such an assumption does not hold when the observations of interest are measures of correlation; indeed, if genes A and B are highly correlated, and genes B and C are highly correlated, then genes A and C are also likely to be highly correlated. Therefore, to control the type I error rate, we performed simulations under the null hypothesis and with varying the parameter, γ. Our results suggest that the γ required to keep the type I error rate at 0.05 increases as a function of M and plateaus approximately 3.1 (Supplementary Methods and Data). For this reason, we decided to adopt *D*_0.05_ with γ(0.05) = 3.1 as the critical value of *D* in this work.

The optimal threshold dichotomizes the gene pairs into strong and weak co-expression classes for both states. The numbers of strongly and weakly co-expressed gene pairs in the neoplastic state are denoted by Q_s,d_ and Q_w,d_, respectively, while those in normal state are Q_s,n_ and Q_w,n_. The association between the co-expression classes and the states is quantified by the log odds ratio:
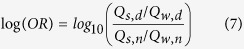
The value of log(OR) follows a normal distribution with a standard error (SE) given by the following formula^28^:
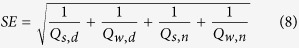


To examine whether the population mean of log(OR) is zero, the value of z-score is obtained by log(OR)/SE, and the p-value is obtained from the area under the two tails of the normal curve delimited by the z-score. When the strong co-expression is associated with a state, it is important to identify the neoplasm-specific, normal-specific, opposing and conforming doublets. We describe the classification in greater detail below.

### Doublet classification

The co-expression galaxy is a scatter plot of the correlation coefficient r_ij_ in the normal state vs. that in the neoplastic state (Supplementary Fig. 2). The optimal threshold, Ĉ, partitions the co-expression galaxy into nine regions. Normal-specific, neoplasm-specific, conforming and opposing doublets reside in the bordering regions, while weakly co-expressed pairs (WCPs), pairs of genes that exhibit co-expression levels below the threshold in both states, reside in the central region. The gene expression levels of a conforming doublet are either positively or negatively correlated in both states. The sign of the correlation of an opposing doublet in one state is the opposite of that in the other state. Genes of a normal-specific doublet are strongly co-expressed in the normal state, but they are weakly co-expressed in the neoplastic state. The genes of a neoplasm-specific doublet are strongly co-expressed in the neoplastic state, but they are weakly co-expressed in the normal state.

To verify the connection of *NPM1* with the known MAPK and PI3K/AKT pathways in CML, the normal- and CML-specific doublets between *NPM1* and the pathway members (*NPM1*-doublets) were extracted from the corresponding regions of the co-expression galaxy. The normal- and CML-specific *NPM1*-doublets were compared to explore the role of *NPM1* in the pathways in CML.

### *NPM1*-related co-expression networks

In addition to the genome-wide analysis of structural difference in co-expression, another important research question is whether the normal and neoplastic states exhibit different co-expression patterns over a set of genes closely related to a particular physiological function or pathological feature. Following the same structural analysis and doublet classification approach mentioned above, the gene pairs were classified into two co-expression classes, and their associations with normal and neoplastic states were quantified by the value of log(OR). The doublets specifically found in the normal state represent the gene-gene associations, e.g., protein-protein interactions, which maintain the physiological function or inhibit the pathological features in the normal state, but they are lost, impaired or bypassed in the neoplastic state. The pathologically altered gene-gene associations represented by the neoplasm-specific doublets indicate the plasticity of the cellular responses to genetic variations or external stress.

According to the gene list curated by Brentani *et al*^29^, *NPM1* is one of 380 cancer-associated genes. In a multiclass cancer study, the global cancer map compendium was derived by the multiclass clustering of the tumor gene expression data, and a set of *NPM1*-associated genes was identified with the criteria that genes with a Pearson correlation no less than 0.8 be included and that the set contains no fewer than 25 genes^14^. We did not apply the same pre-defined threshold in our structural analysis. With 116 total genes, including *NPM1*, the *NPM1*-associated gene set (GCM_NPM1) is stored in the Molecular Signature Database (MSigDB)^14^. Ninety-three of the 116 genes can be found in our microarray dataset. Therefore, the expression profiles of these 93 genes were extracted from the expression matrices for the co-expression analysis of the *NPM1*-associated gene set. The reduced expression matrices have dimension 93 × 8 and 93 × 9, where each row represents the relative expression intensities of a gene across the samples of the same state. The co-expression levels of all 4278 possible gene pairs were computed for each of the normal and CML states.

Using the same approach as the genome-wide analysis, the co-expression galaxy of the *NPM1*-associated gene set was also partitioned into normal-specific, neoplasm-specific, conforming and opposing doublets and WCPs. The gene networks of normal-specific and CML-specific doublets were constructed to help visualize and elucidate the mechanisms underlying the neoplastic pathology and normal physiology related to *NPM1*. From there, we chose to focus on the connections between *NPM1* and its strongly co-expressed genes, termed *NPM1*-doublets, as well as connections among the RP genes, termed RP-doublets, to elucidate the altered association of *NPM1* with ribosome biogenesis in CML.

To visualize the gene networks, we used nodes to represent the individual genes and connections between nodes to indicate that the gene pairs are strongly correlated. The statistical significance of an individual connection was examined using the one-sample t-test based on the following Fisher transformation of *r* to Student’s t-distribution^30^.
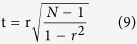
where N is the number of samples for a state and r is the correlation coefficient. To control the expected proportion of false positives, the FDRs of connections were calculated using the Benjamini-Hochberg algorithm based on the t-test p-value^31^. However, this work aimed to discover a set of connections whose synergistic perturbation signifies their structural cooperativity in the disease state compared with the normal state. The paired t-test is reliable for examining such structural perturbations in the gene pair correlations^32^. Before the paired t-test, we obtained the connections’ z-scores for the disease and normal states, respectively, based on the following Fisher transformation of r to a normal distribution.
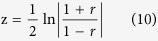


The paired differences are given by,
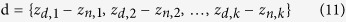
where *z*_*d,i*_ and *z*_*n,i*_ are the Fisher-transforms of *r* of the *i*^th^ connection in CML and normal states, respectively, and k is the total number of connections in the network.

The t statistic is given by,
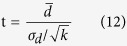
where 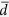 and 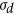 are, respectively, the mean and standard deviation of d over all of the connections in the network. A p-value was obtained to indicate the overall significance of the identified network.

Furthermore, the identified connections were validated by cell line experiments and their underlying mechanisms were elucidated using functional annotation analysis.

### Cell line experiment

Based on the co-expression analysis, gene pairs were classified into normal-specific, CML-specific, opposing and conforming doublets. We focused on CML-specific *NPM1*-doublets and investigated their expression levels in CML cells under resveratrol treatment, which is a known potent anti-inflammatory agent that is often applied in anti-cancer treatment with other therapeutic anti-cancer drugs^33^,^34^. K562 cells, a human CML cell line, were grown in RPMI1640 medium supplemented with 10% fetal bovine serum. Cultures were incubated at 37 °C in a humidified 5% CO_2_ incubator. To validate the co-expression network, K562 cells were treated for 24 hours with 30 μM Resveratrol (Res) (Sigma-Aldrich, MO, USA) or with DMSO (Sigma-Aldrich, MO, USA) as a vehicle control. Then, the K562 cells were collected and harvested for total RNA extraction.

Total RNA was isolated from control- or Res-treated K562 cells using the Trizol Reagent (Life Technologies, Thermo Fisher Scientific, MA, USA) according to manufacturer’s protocol. Following RNA extraction, 2 μg of total RNA was reverse-transcribed cDNA with oligo (dT) 15 using M-MLV reverse transcriptase (Life Technologies, Thermo Fisher Scientific, MA, USA) in a total volume of 20 μL of reactive volume. After reverse transcription reaction, each cDNA sample was diluted by DEPC-treated H_2_O in a final volume of 40 μL/sample and stored at −20 °C or immediately used for real-time PCR.

Twenty-one genes, which were found by our structural analysis to be strongly co-expressed with *NPM1* in CML-specific networks, were selected for validation. Real-time PCR was performed using Maxima^TM^ SYBR Green/ROX qPCR Master Mix (Fermantas, Thermo Fisher Scientific, MA, USA) and ABI Prism 7500 system (Applied Biosystems, Thermo Fisher Scientific, MA, USA). The primer sequences used in real-time PCR are listed in Supplementary Table 9. Triplicate PCR experiments were performed. All data were analyzed after normalizing to the β-actin expression values of the respective sample, and the expression levels are presented by the mean ±SD of at least three independent experiments.

### Functional annotation analysis

The co-expression network analysis of *NPM1*-related genes identified five mutually exclusive networks, including the CML-specific, opposing, normal-specific, conforming, and weak co-expression networks. To elucidate the biological roles and pathways of these networks, functional annotation analysis was performed on these networks using three collections of predefined functional gene set databases. The three collections are the GeneSetDB^18^, Reactome pathway database^20^, and Molecular Signatures Database (MSigDB, v3.0)^14^. Together, they provide 2,431 GO sets, 1,345 Reactome pathways (as of Oct 12, 2012) and 186 KEGG pathways.

Conventional gene set analysis uses single genes as basic items for mapping between the experimentally identified genes and a functional gene set^14^. However, the basic items of co-expression network are gene pairs so that the conventional approach cannot address the connectivity of genes through the mapping of individual genes. We developed a pair-based mapping approach for the functional annotation of the identified networks. A gene pair in the identified network was mapped onto a functional gene set if both of the genes of the pair were found in the gene set. After the pair-based mapping, two-by-two contingency tables were formed for which gene pairs were classified according to two criteria (Supplementary Table 10). The first criterion was whether both genes in a pair were found in the gene set. The second criterion was whether the gene pairs were from a particular network (e.g., CML-specific) or whether they were from one of the other four networks (e.g., opposing, normal-specific, conforming, or weak). In each, *H* denotes the total number of all possible gene pairs, *h* the number of gene pairs in a particular network, *K* the number of gene pairs found in the functional gene set, and *k* the number of gene pairs that are in both the network and functional gene set.

Finally, a two-tailed Fisher’s exact test was performed to determine whether the gene pairs of a network are significantly associated with a gene set^35^. Under the null hypothesis, the network and functional gene set are independent. Therefore, based on the hyper-geometric distribution, the probability *p*_*k*_ of observing a particular 2 × 2 table under the null hypothesis is calculated as follows:
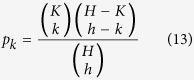


The totals along the rows and columns, i.e., *K, H-K, h* and *H-h* in Supplementary Table 10, are known as the marginal totals. With the same marginal totals, there may be some other possible combinations of the four entries in the contingency table, and each combination is accompanied with a probability *p*_*i*_. By fixing the marginal totals as those of the observed outcomes, the *p*-value for testing the null hypothesis was calculated by summing the probabilities of combinations, *p*_*i*_’s, that are less than or equal to the probability *p*_*k*_ of the observed outcomes^36^,^37^. The formula for the *p*-value is then defined as follows:
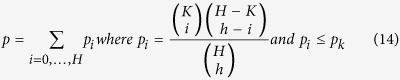


The *p*-values were computed for all possible mappings between the five identified networks and functional gene sets. Functional gene sets without any gene pair found in the identified networks (i.e., *k* = 0) were excluded from the test because of the lack of information for evaluating their associations with the networks. The computed *p*-values were then adjusted for multiple testing using the Benjamini and Hochberg’s method^6^ and Bonferroni correction^38^. The adjustment was performed independently for different networks and different gene set collections.

The Fisher’s exact test examines the significance of the association between a network and functional gene set. To determine whether the network is over-represented or under-represented in the functional gene set, we compared the observed number of gene pairs of the network found in the functional gene set, *k*, with its expected value, *k*_*e*_. Under the null hypothesis, *k*_*e*_ can be estimated using the marginal totals of the contingency table as follows:
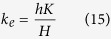
Therefore, if *k* is greater than *k*_*e*_, the network is over-represented in the gene set. On the other hand, if *k* is less than *k*_*e*_, the network is under-represented.

### Cooperativities of transcription factors

Two genes tend to be co-expressed when they are regulated by the same TFs^39^. We compared the CML-specific and normal-specific *NPM1*-doublets with respect to the TFs that concurrently target them. We hypothesized that the TFs may drive the neoplastic alteration of the co-expression patterns. The potential TFs of the doublets were identified by searching the prediction of the transcriptional regulatory modules (PReMod) database^15^. The roles of the TFs on the *NPM1*-doublets were investigated to gain insight into the role of transcriptional regulation in the *NPM1*-oriented molecular mechanism of CML.

## Additional Information

**How to cite this article**: Chan, L. W. C. *et al.* Novel structural co-expression analysis linking the NPM1-associated ribosomal biogenesis network to chronic myelogenous leukemia. *Sci. Rep.* **5**, 10973; doi: 10.1038/srep10973 (2015).

## Supplementary Material

Supplementary Information
