# Supplementary material for: Novel structural co-expression analysis linking the NPM1-associated ribosomal biogenesis network to chronic myelogenous leukemia
Source: Sci Rep. 2015 Jul 24;5:10973. doi: 10.1038/srep10973 (PMC4513283; doi:10.1038/srep10973)
Supplement: Supplementary Information [file srep10973-s1.doc]

**Supplementary Materials**

**Novel structural co-expression analysis linking the *NPM1*-associated ribosomal biogenesis network to chronic myelogenous leukemia**

Lawrence WC Chan^1^, Xihong Lin^2^, Godwin Yung^2^, Thomas Lui^1^, Ya Ming Chiu^1^, Fengfeng Wang^1^, Nancy BY Tsui^1^, William CS Cho^3^, SP Yip^1^, Parco M Siu^1^, SC Cesar Wong^1^, Benjamin YM Yung^1*^

^1^Department of Health Technology and Informatics, The Hong Kong Polytechnic University, Hong Kong; ^2^Department of Biostatistics, School of Public Health, Harvard University, Massachusetts, USA; ^3^Department of Clinical Oncology, Queen Elizabeth Hospital, Hong Kong


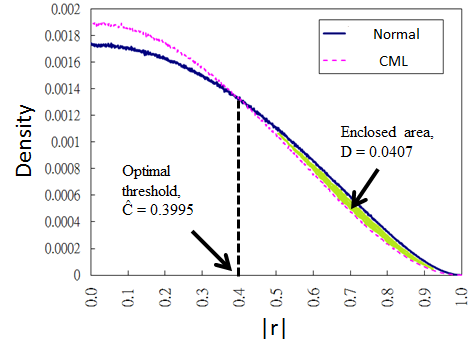


**Supplementary Fig. 1** Transcriptome-wide co-expression structures of normal state samples (blue solid line) and CML samples (red dotted line). The crossover point indicates the optimal threshold at Ĉ = 0.3995. The green shaded area enclosed by the two distribution curves represents the D statistic of the two-sample KS test where D = 0.0407 (D_0.05_ = 1.7016×10^-7^).


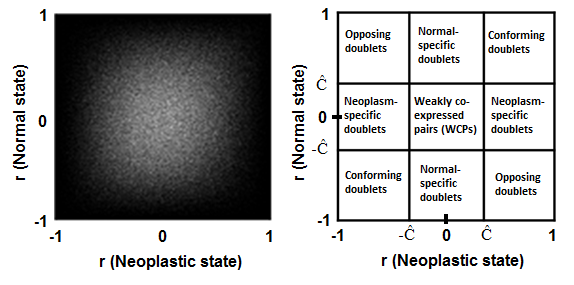


**Supplementary Fig. 2** Co-expression galaxy (left) and nine regions partitioned by optimal threshold Ĉ (right).


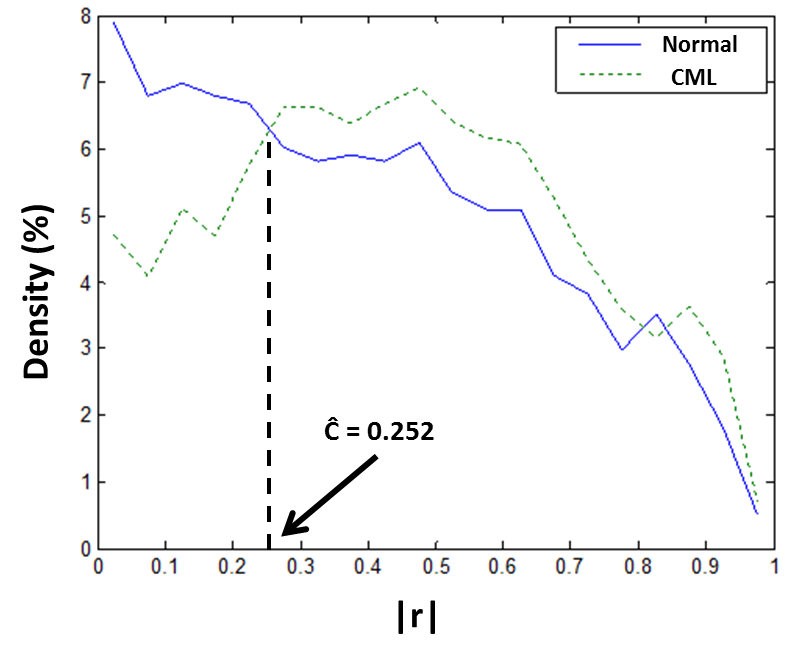


**Supplementary Fig. 3** *NPM1*-related co-expression structures of normal state samples (blue solid line) and CML samples (green dotted line). The crossover point indicates the optimal threshold at Ĉ = 0.252 where D = 0.1087 (D_0.05_ = 0.067).


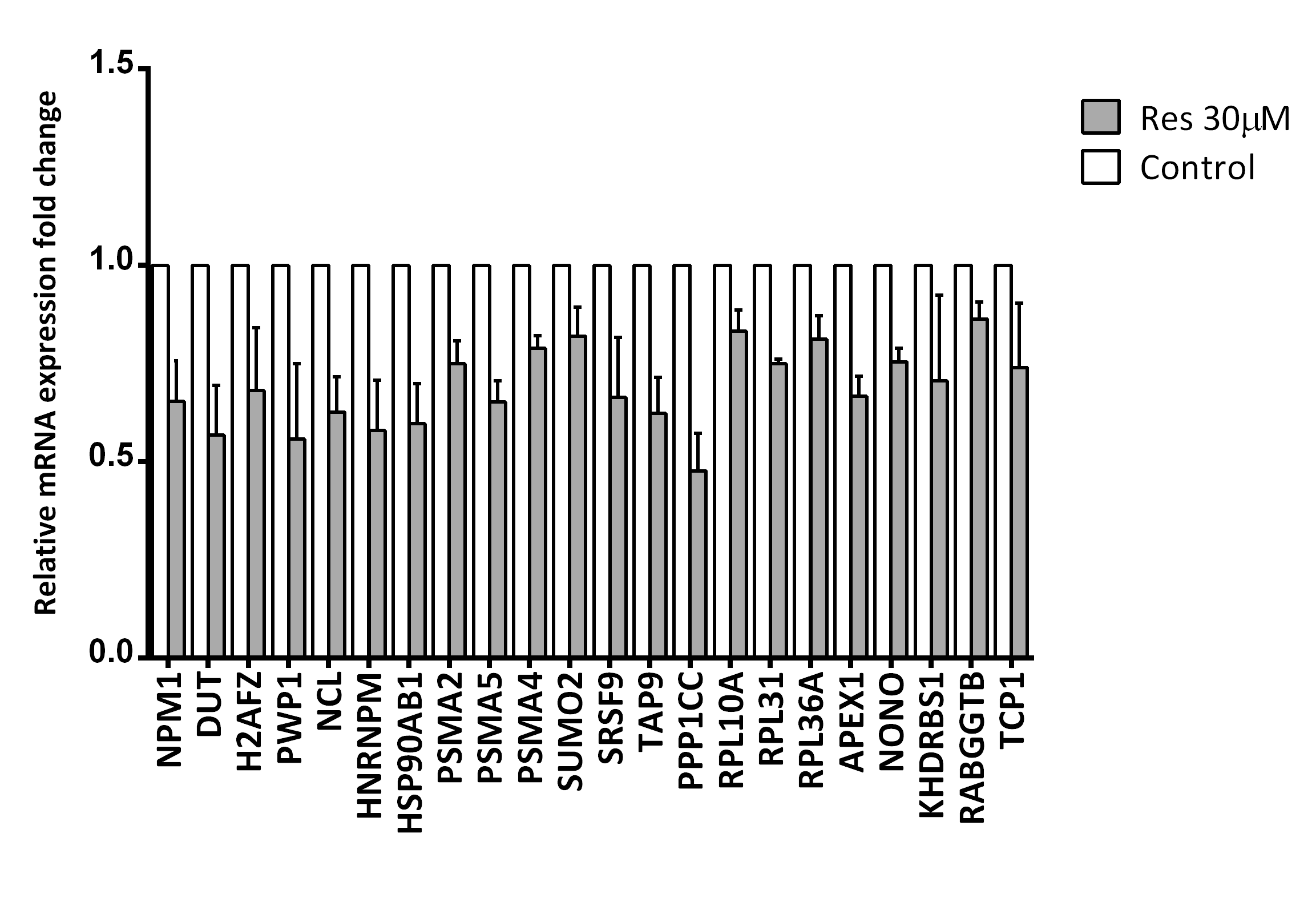


**Supplementary Fig. 4** Resveratrol (Res)-mediated repression of *NPM1* and its co-expressed RNAs in K562 cells as quantified by real-time RT-PCR. For each transcript, the expression levels were normalized such that the levels of DMSO-treated (control) cells are displayed as 1.0. Means ± standard deviations of three independent experiments were shown for Res-treatment groups. Significant reductions of expression levels after Res-treatment were found for all of the 21 mRNAs together with *NPM1* (t-test, *P* < 0.05).


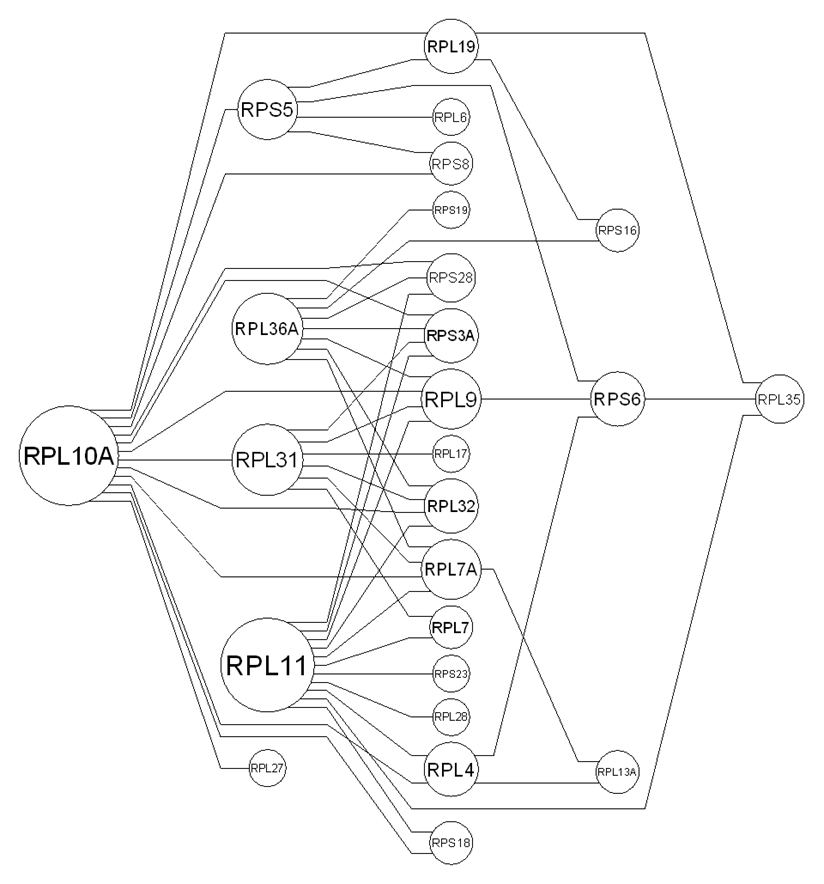


**Supplementary Fig. 5** CML-specific network map of the “KEGG Ribosome” gene set.

**Supplementary Fig 6.** CML-specific network of “Disease” gene set in Reactome.


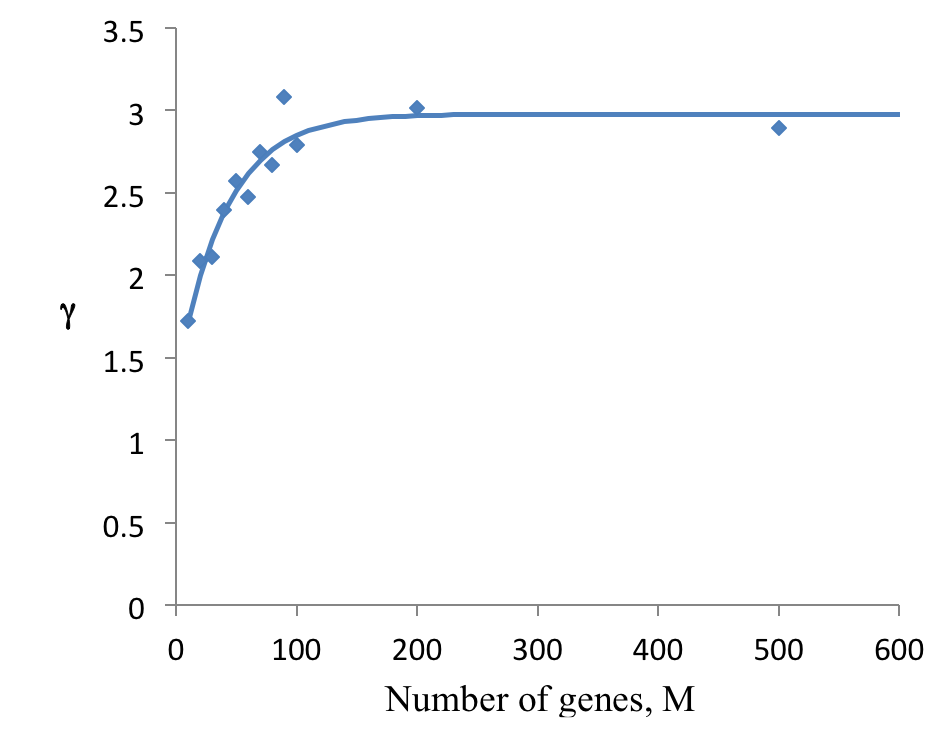


**Supplementary Fig 7.** Plot of coefficient γ against gene number M.

**Supplementary Table 1.** Cross-tabulation of gene pair counts in genome-wide analysis.

| **Class\State**  **(million pairs)** | **CML** | **Normal** |
| --- | --- | --- |
| **Strong**  **co-expression** | 11.757 | 23.198 |
| **Weak**  **co-expression** | 24.679 | 13.238 |

**Supplementary Table 2.** Cross-tabulation of gene pair counts in NPM1-related co-expression analysis.

| **Class\State** | **CML** | **Normal** |
| --- | --- | --- |
| **Strong**  **co-expression** | 3228 | 2763 |
| **Weak**  **co-expression** | 1050 | 1515 |

**Supplementary Table 3**. Correlation test results of individual CML-specific doublets.

| **Gene i** | **Gene j** | **r (CML)** | **t** | **p** | **FDR** |
| --- | --- | --- | --- | --- | --- |
| **RPL13A** | **RPL7A** | 0.931357 | 6.767614 | 0.000261 | 0.0027 |
| **RPL13A** | **RPL4** | 0.928959 | 6.63942 | 0.000293 | 0.0015 |
| **RPL11** | **RPL32** | 0.912768 | 5.912069 | 0.000592 | 0.002 |
| **RPL9** | **RPS6** | 0.906176 | 5.669214 | 0.000759 | 0.0019 |
| **RPL11** | **RPL9** | 0.896486 | 5.353258 | 0.00106 | 0.0022 |
| **RPL10A** | **RPS5** | 0.890787 | 5.186383 | 0.001272 | 0.0022 |
| **NPM1** | **RPL36A** | 0.883995 | 5.002896 | 0.00156 | 0.0023 |
| **RPL36A** | **RPL7A** | 0.880023 | 4.902438 | 0.001748 | 0.0022 |
| **RPL36A** | **RPS28** | 0.874141 | 4.761986 | 0.002055 | 0.0023 |
| **RPL36A** | **RPS3A** | 0.871921 | 4.711334 | 0.002179 | 0.0022 |
| **RPL32** | **RPL36A** | 0.866853 | 4.600176 | 0.002484 | 0.0023 |
| **RPL36A** | **RPL9** | 0.863276 | 4.525198 | 0.002715 | 0.0023 |
| **RPL11** | **RPL35** | 0.85659 | 4.392127 | 0.003187 | 0.0025 |
| **RPL11** | **RPS18** | 0.841772 | 4.125572 | 0.004429 | 0.0032 |
| **RPL11** | **RPL7A** | 0.835221 | 4.01842 | 0.00507 | 0.0035 |
| **RPL4** | **RPS6** | 0.824979 | 3.862048 | 0.006195 | 0.004 |
| **RPL11** | **RPL4** | 0.819288 | 3.780431 | 0.006888 | 0.0042 |
| **RPL36A** | **RPS16** | 0.819046 | 3.777041 | 0.006919 | 0.0039 |
| **RPL19** | **RPS16** | 0.813856 | 3.705705 | 0.007598 | 0.0041 |
| **RPL11** | **RPS3A** | 0.762469 | 3.117814 | 0.016895 | 0.0087 |
| **RPL31** | **RPL32** | 0.754036 | 3.03729 | 0.018919 | 0.0092 |
| **RPL11** | **RPS28** | 0.746289 | 2.966387 | 0.020915 | 0.0098 |
| **RPL11** | **RPL7** | 0.741771 | 2.926318 | 0.02214 | 0.0099 |
| **H2AFZ** | **NPM1** | 0.720862 | 2.751809 | 0.02843 | 0.0122 |
| **HNRNPM** | **NPM1** | 0.714634 | 2.702998 | 0.030506 | 0.0125 |
| **HSP90AB1** | **NPM1** | 0.713883 | 2.697202 | 0.030763 | 0.0121 |
| **RPL36A** | **RPS19** | 0.707103 | 2.645723 | 0.033147 | 0.0126 |
| **RPL10A** | **RPS14** | 0.706787 | 2.643356 | 0.033261 | 0.0122 |
| **NONO** | **NPM1** | 0.689329 | 2.517494 | 0.039957 | 0.0141 |
| **RPL11** | **RPS23** | 0.680164 | 2.454842 | 0.043795 | 0.015 |
| **NPM1** | **TAF9** | 0.677317 | 2.435819 | 0.045034 | 0.0149 |
| **RPL31** | **RPL9** | 0.656533 | 2.302839 | 0.054762 | 0.0176 |
| **RPL11** | **RPL28** | 0.653094 | 2.281761 | 0.056489 | 0.0176 |
| **RPL17** | **RPL31** | 0.649324 | 2.258936 | 0.058423 | 0.0176 |
| **RPL10A** | **RPL31** | 0.648788 | 2.25572 | 0.058701 | 0.0172 |
| **NPM1** | **PSMA5** | 0.642562 | 2.218724 | 0.061994 | 0.0177 |
| **RPL10A** | **RPL27** | 0.631878 | 2.15696 | 0.067912 | 0.0188 |
| **RPL35** | **RPS6** | 0.627117 | 2.130112 | 0.070659 | 0.0191 |
| **NPM1** | **TCP1** | 0.591797 | 1.942403 | 0.093205 | 0.0245 |
| **DUT** | **NPM1** | 0.570707 | 1.838813 | 0.108534 | 0.0278 |
| **RPL10A** | **RPL32** | 0.559468 | 1.785862 | 0.117285 | 0.0294 |
| **RPL19** | **RPL35** | 0.55778 | 1.778034 | 0.118635 | 0.029 |
| **RPL19** | **RPS5** | 0.544669 | 1.718304 | 0.129436 | 0.0309 |
| **RPL10A** | **RPL7A** | 0.541429 | 1.703829 | 0.132191 | 0.0308 |
| **RPL31** | **RPL7** | 0.536065 | 1.680091 | 0.136829 | 0.0312 |
| **RPL6** | **RPS5** | 0.528859 | 1.648655 | 0.143208 | 0.0319 |
| **NCL** | **NPM1** | 0.522328 | 1.62059 | 0.149137 | 0.0326 |
| **NPM1** | **PSMA2** | 0.52005 | 1.610894 | 0.151238 | 0.0323 |
| **RPL10A** | **RPL4** | 0.511576 | 1.575235 | 0.159205 | 0.0333 |
| **RPL31** | **RPL7A** | 0.507824 | 1.559648 | 0.162808 | 0.0334 |
| **RPS5** | **RPS8** | 0.489821 | 1.486476 | 0.180744 | 0.0364 |
| **RPL31** | **RPS3A** | 0.465509 | 1.391594 | 0.206665 | 0.0408 |
| **RPS5** | **RPS6** | 0.462605 | 1.380541 | 0.209891 | 0.0406 |
| **RPL10A** | **RPS28** | 0.459209 | 1.367684 | 0.2137 | 0.0406 |
| **NPM1** | **PWP1** | 0.453975 | 1.34802 | 0.219642 | 0.041 |
| **RPL10A** | **RPL9** | 0.41228 | 1.19728 | 0.270165 | 0.0495 |
| **RPL10A** | **RPS8** | 0.409178 | 1.186452 | 0.274146 | 0.0494 |
| **KHDRBS1** | **NPM1** | 0.407015 | 1.178931 | 0.276941 | 0.049 |
| **RPL10A** | **RPS18** | 0.363837 | 1.033451 | 0.33577 | 0.0584 |
| **NPM1** | **RABGGTB** | 0.362288 | 1.028386 | 0.337986 | 0.0578 |
| **NPM1** | **PPP1CC** | 0.346992 | 0.978875 | 0.360252 | 0.0606 |
| **NPM1** | **RPL10A** | 0.341869 | 0.962493 | 0.367864 | 0.0609 |
| **NPM1** | **SRSF9** | 0.329278 | 0.92264 | 0.386891 | 0.063 |
| **NPM1** | **PSMB4** | 0.329236 | 0.922508 | 0.386955 | 0.062 |
| **RPL10A** | **RPS3A** | 0.327652 | 0.917534 | 0.389381 | 0.0615 |
| **NPM1** | **RPL31** | 0.31984 | 0.893131 | 0.401447 | 0.0624 |
| **RPL10A** | **RPL19** | 0.314147 | 0.875477 | 0.410347 | 0.0628 |
| **APEX1** | **NPM1** | 0.30822 | 0.857207 | 0.419707 | 0.0633 |
| **NPM1** | **SUMO2** | 0.276877 | 0.762351 | 0.470753 | 0.07 |

**Supplementary Table 4**. Comparison of co-expression perturbation between CML and normal over all the CML-specific doublets.

| **Gene i** | **Gene j** | **r (Normal)** | **r (CML)** | **z (Normal)** | **z (CML)** | **z (CML) – z (Normal)** |
| --- | --- | --- | --- | --- | --- | --- |
| **RPL13A** | **RPL7A** | 0.192918 | 0.931357 | 0.195367 | 1.668529 | 1.473163 |
| **RPL13A** | **RPL4** | 0.239008 | 0.928959 | 0.243722 | 1.650741 | 1.407019 |
| **RPL11** | **RPL32** | 0.05469 | 0.912768 | 0.054744 | 1.543866 | 1.489122 |
| **RPL9** | **RPS6** | 0.223982 | 0.906176 | 0.227844 | 1.505716 | 1.277872 |
| **RPL11** | **RPL9** | 0.010834 | 0.896486 | 0.010834 | 1.454026 | 1.443192 |
| **RPL10A** | **RPS5** | 0.233709 | 0.890787 | 0.238109 | 1.425725 | 1.187616 |
| **NPM1** | **RPL36A** | 0.219947 | 0.883995 | 0.2236 | 1.39376 | 1.17016 |
| **RPL36A** | **RPL7A** | 0.159316 | 0.880023 | 0.160685 | 1.375869 | 1.215184 |
| **RPL36A** | **RPS28** | 0.223142 | 0.874141 | 0.22696 | 1.350374 | 1.123413 |
| **RPL36A** | **RPS3A** | 0.230224 | 0.871921 | 0.234426 | 1.341037 | 1.106611 |
| **RPL32** | **RPL36A** | 0.033908 | 0.866853 | 0.033921 | 1.320279 | 1.286359 |
| **RPL36A** | **RPL9** | 0.250054 | 0.863276 | 0.25547 | 1.306063 | 1.050593 |
| **RPL11** | **RPL35** | 0.204083 | 0.85659 | 0.206989 | 1.280396 | 1.073407 |
| **RPL11** | **RPS18** | 0.132297 | 0.841772 | 0.133077 | 1.227224 | 1.094147 |
| **RPL11** | **RPL7A** | 0.00161 | 0.835221 | 0.00161 | 1.205157 | 1.203548 |
| **RPL4** | **RPS6** | 0.17611 | 0.824979 | 0.177965 | 1.172208 | 0.994244 |
| **RPL11** | **RPL4** | 0.09262 | 0.819288 | 0.092887 | 1.154648 | 1.061761 |
| **RPL36A** | **RPS16** | 0.227364 | 0.819046 | 0.231408 | 1.153913 | 0.922505 |
| **RPL19** | **RPS16** | 0.178419 | 0.813856 | 0.18035 | 1.138345 | 0.957996 |
| **RPL11** | **RPS3A** | 0.093063 | 0.762469 | 0.093333 | 1.002086 | 0.908753 |
| **RPL31** | **RPL32** | -0.18221 | 0.754036 | -0.18427 | 0.982245 | 1.166514 |
| **RPL11** | **RPS28** | 0.130622 | 0.746289 | 0.131372 | 0.964525 | 0.833153 |
| **RPL11** | **RPL7** | 0.228328 | 0.741771 | 0.232425 | 0.954407 | 0.721982 |
| **H2AFZ** | **NPM1** | 0.157332 | 0.720862 | 0.15865 | 0.909436 | 0.750787 |
| **HNRNPM** | **NPM1** | -0.09776 | 0.714634 | -0.09807 | 0.896591 | 0.994665 |
| **HSP90AB1** | **NPM1** | -0.12468 | 0.713883 | -0.12533 | 0.895058 | 1.020393 |
| **RPL36A** | **RPS19** | 0.111831 | 0.707103 | 0.112301 | 0.881366 | 0.769065 |
| **RPL10A** | **RPS14** | 0.162623 | 0.706787 | 0.164079 | 0.880733 | 0.716654 |
| **NONO** | **NPM1** | 0.039681 | 0.689329 | 0.039701 | 0.846677 | 0.806975 |
| **RPL11** | **RPS23** | 0.129806 | 0.680164 | 0.130543 | 0.82942 | 0.698877 |
| **NPM1** | **TAF9** | -0.1359 | 0.677317 | -0.13675 | 0.824139 | 0.960888 |
| **RPL31** | **RPL9** | -0.20192 | 0.656533 | -0.20473 | 0.786696 | 0.991431 |
| **RPL11** | **RPL28** | 0.179291 | 0.653094 | 0.181251 | 0.780675 | 0.599425 |
| **RPL17** | **RPL31** | -0.14217 | 0.649324 | -0.14314 | 0.774128 | 0.91727 |
| **RPL10A** | **RPL31** | -0.0428 | 0.648788 | -0.04283 | 0.773204 | 0.816033 |
| **NPM1** | **PSMA5** | 0.107685 | 0.642562 | 0.108104 | 0.762526 | 0.654422 |
| **RPL10A** | **RPL27** | 0.214876 | 0.631878 | 0.218278 | 0.744536 | 0.526258 |
| **RPL35** | **RPS6** | 0.163039 | 0.627117 | 0.164508 | 0.736651 | 0.572143 |
| **NPM1** | **TCP1** | -0.13603 | 0.591797 | -0.13688 | 0.680426 | 0.817309 |
| **DUT** | **NPM1** | 0.030961 | 0.570707 | 0.030971 | 0.648571 | 0.6176 |
| **RPL10A** | **RPL32** | -0.00155 | 0.559468 | -0.00155 | 0.632059 | 0.633605 |
| **RPL19** | **RPL35** | 0.240385 | 0.55778 | 0.245183 | 0.629605 | 0.384423 |
| **RPL19** | **RPS5** | 0.213077 | 0.544669 | 0.216392 | 0.61077 | 0.394378 |
| **RPL10A** | **RPL7A** | 0.239541 | 0.541429 | 0.244287 | 0.606176 | 0.361888 |
| **RPL31** | **RPL7** | -0.22984 | 0.536065 | -0.23402 | 0.598617 | 0.832636 |
| **RPL6** | **RPS5** | 0.226519 | 0.528859 | 0.230517 | 0.58856 | 0.358042 |
| **NCL** | **NPM1** | -0.09657 | 0.522328 | -0.09687 | 0.579536 | 0.676409 |
| **NPM1** | **PSMA2** | -0.14991 | 0.52005 | -0.15105 | 0.576408 | 0.727456 |
| **RPL10A** | **RPL4** | 0.231176 | 0.511576 | 0.235431 | 0.564862 | 0.32943 |
| **RPL31** | **RPL7A** | -0.10998 | 0.507824 | -0.11043 | 0.559793 | 0.670222 |
| **RPS5** | **RPS8** | 0.240257 | 0.489821 | 0.245047 | 0.535825 | 0.290778 |
| **RPL31** | **RPS3A** | -0.19506 | 0.465509 | -0.19759 | 0.504321 | 0.701909 |
| **RPS5** | **RPS6** | 0.223051 | 0.462605 | 0.226864 | 0.500621 | 0.273757 |
| **RPL10A** | **RPS28** | 0.155688 | 0.459209 | 0.156964 | 0.496308 | 0.339344 |
| **NPM1** | **PWP1** | -0.23025 | 0.453975 | -0.23446 | 0.489696 | 0.724154 |
| **RPL10A** | **RPL9** | 0.227746 | 0.41228 | 0.231811 | 0.438355 | 0.206544 |
| **RPL10A** | **RPS8** | 0.12204 | 0.409178 | 0.122652 | 0.434624 | 0.311972 |
| **KHDRBS1** | **NPM1** | 0.045161 | 0.407015 | 0.045192 | 0.432028 | 0.386837 |
| **RPL10A** | **RPS18** | 0.010827 | 0.363837 | 0.010827 | 0.381301 | 0.370474 |
| **NPM1** | **RABGGTB** | -0.23473 | 0.362288 | -0.23919 | 0.379517 | 0.61871 |
| **NPM1** | **PPP1CC** | -0.18135 | 0.346992 | -0.18338 | 0.36202 | 0.545398 |
| **NPM1** | **RPL10A** | 0.204929 | 0.341869 | 0.207872 | 0.356207 | 0.148335 |
| **NPM1** | **SRSF9** | -0.14426 | 0.329278 | -0.14527 | 0.342018 | 0.48729 |
| **NPM1** | **PSMB4** | 0.075816 | 0.329236 | 0.075962 | 0.341971 | 0.266009 |
| **RPL10A** | **RPS3A** | 0.213097 | 0.327652 | 0.216413 | 0.340195 | 0.123782 |
| **NPM1** | **RPL31** | -0.15293 | 0.31984 | -0.15414 | 0.331469 | 0.485608 |
| **RPL10A** | **RPL19** | 0.054779 | 0.314147 | 0.054834 | 0.32514 | 0.270306 |
| **APEX1** | **NPM1** | -0.03304 | 0.30822 | -0.03305 | 0.318577 | 0.351627 |
| **NPM1** | **SUMO2** | -0.03188 | 0.276877 | -0.03189 | 0.284297 | 0.316185 |

**Supplementary Table 5.** Significantly associated functional annotations of Gene Ontology (Bonferroni adjusted *p*-value < 0.05).

Note:

1. The third column is the GO categories including 1) BP: biological process, 2) MF: molecular function, and 3) CC: cellular component.
2. *k* is the number of gene pairs of the network found in the gene set.
3. *k_e_* is the expected number of gene pairs found in the gene set.
4. FDR and Bonferroni adjusted *p*-value are highlighted if less than 0.05.
5. Gene sets are sorted according to their *p*-values to CML-specific network.

**Supplementary Table 6.** Significantly associated functional annotations in Reactome pathway (Bonferroni adjusted *p*-value < 0.05).

Note:

1. *k* is the number of gene pairs of the network found in the gene set.
2. *k_e_* is the expected number of gene pairs found in the gene set.
3. FDR and Bonferroni adjusted *p*-value are highlighted if less than 0.05.
4. Gene sets are sorted according to their *p*-values to CML-specific network.

**Supplementary Table 7.** Significantly associated functional annotations in KEGG pathways (Bonferroni adjusted *p*-value < 0.05).

Note:

1. *k* is the number of gene pairs of the network found in the gene set.
2. *k_e_* is the expected number of gene pairs found in the gene set.
3. FDR and Bonferroni adjusted *p*-value are highlighted if less than 0.05.
4. Gene sets are sorted according to their *p*-values to CML-specific network.

**Supplementary Table 8.** Transcription factors targeting *NPM1*-doublets in CML- and normal-specific co-expression networks. Transcription factors shared between the two networks are bolded.

1. CML-specific *NPM1*-doublets

|  | **CML-specific doublets** | | **Correlation coefficient** |
| --- | --- | --- | --- |
| **Transcription Factors** | **Target 1** | **Target 2** | **r (CML)** |
| **E2F1, E2F3, E2F4, TFDP1** | *APEX1* | *NPM1* | **0.308** |
| CEBPA | *DUT* | *NPM1* | **0.571** |
| **E2F1, E2F3, E2F4, MAX, MYC, TFDP1** | *H2AFZ* | *NPM1* | **0.721** |
| **E2F1, E2F3, E2F4, TFDP1, ZEB1**, CEBPA, DDIT3 | *HNRNPM* | *NPM1* | **0.715** |
| **E2F1, E2F3, E2F4, TFDP1** | *HSP90AB1* | *NPM1* | **0.714** |
| **E2F1, E2F3, E2F4, STAT5A, TFDP1** | *KHDRBS1* | *NPM1* | **0.407** |
| - | *NCL* | *NPM1* | **0.522** |
| **E2F1, E2F3, E2F4, TFDP1**, CEBPA, DDIT3, NKX6-1 | *NONO* | *NPM1* | **0.689** |
| **E2F1, E2F3, E2F4, FALZ, TFDP1**, NRF1 | *PPP1CC* | *NPM1* | **0.347** |
| **CREB1**, NRF1 | *PSMA2* | *NPM1* | **0.520** |
| **E2F1, E2F3, E2F4, PAX2, TFDP1** | *PSMA5* | *NPM1* | **0.643** |
| **E2F1, E2F3, E2F4, TFDP1**, ATF6, TFAP2A, TFAP2C | *PSMB4* | *NPM1* | **0.329** |
| ATF6, TBP | *PWP1* | *NPM1* | **0.454** |
| **E2F1, E2F3, E2F4, TFDP1** | *RABGGTB* | *NPM1* | **0.362** |
| - | *RPL10A* | *NPM1* | **0.342** |
| - | *RPL31* | *NPM1* | **0.320** |
| **E2F1, E2F3, E2F4, PAX2, TFDP1** | *RPL36A* | *NPM1* | **0.884** |
| - | *SRSF9* | *NPM1* | **0.329** |
| - | *SUMO2* | *NPM1* | **0.277** |
| - | *TAF9* | *NPM1* | **0.677** |
| **PAX2** | *TCP1* | *NPM1* | **0.592** |

1. Normal-specific NPM1-doublets

|  | **Normal-specific doublets** | | **Correlation coefficient** |
| --- | --- | --- | --- |
| **Transcription Factors** | **Target 1** | **Target 2** | **r (Normal)** |
| **CREB1, E2F1, E2F3, E2F4, TFDP1** | *PRMT1* | *NPM1* | **-0.432** |
| **E2F1, PAX2** | *QARS* | *NPM1* | **-0.385** |
| - | *SET* | *NPM1* | **0.469** |
| **E2F1, E2F3, E2F4, STAT5A, TFDP1, ZEB1** | *TIAL1* | *NPM1* | **0.596** |
| - | *APRT* | *NPM1* | **0.822** |
| **CREB1, E2F1, E2F3, E2F4, FALZ, MAX, MYC, PAX2, STAT5A, TFDP1, ZEB1** | *COL16A1* | *NPM1* | **-0.747** |

**Supplementary Table 9.** Primer sequences for real-time RT-PCR

| **Gene** | **Primer sequence** |
| --- | --- |
| **SUMO2** | 5’-GTTTTCCCTTCAAGTCAAGC-3’ |
|  | 5’-GTTACTTCTAAGCAGGCCTAT-3’ |
| **DUT** | 5’-ACCTATGGAGAAAGCTGTT-3’ |
|  | 5’-TAAAGTGTTTTGCAGCCAAG-3’ |
| **TCP1** | 5’-GCTCGGGTACGCTCCAC-3’ |
|  | 5’-CCATCTTGACGGCAGCGATA-3’ |
| **NONO** | 5’-ACAGGGCCGTGTAGC-3’ |
|  | 5’-CATTTTTGCACCCTCAACTT-3’ |
| **PSMB4** | 5’-TTTCAAACCGCCACTGTCAC-3’ |
|  | 5’-TGGGCAATATCCCAGTTGGT-3’ |
| **PSMA5** | 5’-GGACCTTTGTACAGTGTGAT-3’ |
|  | 5-‘GCTTCTCCTCCATTACTTGT-3’ |
| **PSMA2** | 5’-CTTAATGCCCTCTTCGCTAT-3’ |
|  | 5’-TTCCATGAATCCGTGAAAGT-3’ |
| **PPP1CC** | 5’-AGAAGCCAAATGCCACGAGA-3’ |
|  | 5’-TCACGACTAGGCAGTGTCAA-3’ |
| **PWP1** | 5’-GATTCAGTGGGCAGATAGAG-3’ |
|  | 5’-TATCTGAACGTGCATCCAAA-3’ |
| **NCL** | 5’-GGACAGTAAGAAAGAGCGAG-3’ |
|  | 5’-GCTGACTAATCTGATCTCCG-3’ |
| **KHDRBS1** | 5’-AGGATTCCTGTTGCTTTACC-3’ |
|  | 5’-AAGAATGGAATAAGGTGGGG-3’ |
|  |  |
| **HSP90AB1** | 5’-AAAGCACAACGATGATGAAC-3’ |
|  | 5’-TTCACTACTTCTTTGACCCG-3’ |
| **HNRNPM** | 5’-TGAGAAATCTGCCATTCGAT-3’ |
|  | 5’-ATTCTCCATCTTGATGTCGG-3’ |
| **H2AFZ** | 5’-CTTGCTATTCGTGGAGATGA-3’ |
|  | 5’-TCCCAATCAGAGATTTGTGG-3’ |
| **SRSF9** | 5’-TACCAGGGCCATATTAGCA-3’ |
|  | 5’-CTCCATAGTAGAGCGAACCC-3’ |
| **APEX1** | 5’-AAAATATTGCTTCGGTGGGT-3’ |
|  | 5’-CTTACTCTTCTTGGCCTCTG-3’ |
| **RABGGTB** | 5’-TCCTAAGTCTACCCAGGAAC-3’ |
|  | 5’-CAATAACAAAGTGTCCGGTG-3’ |
| **TAF9** | 5’-CCATCATTAATCGGGTCCAA-3’ |
|  | 5’-ACATTCAGCAAGGCTAGATT-3’ |
| **RPL36A** | 5’-AAGATTGTGCTAAGGCTTGA-3’ |
|  | 5’-TTAGAACTGGATCACTTGGC-3’ |
| **RPL31** | 5’-CCGAGAATACACCATCAACA-3’ |
|  | 5’-CGGTATGGCACATTCCTTAT-3’ |
| **RPL10A** | 5’-ATGAGGTGAAGTCCACAATC-3’ |
|  | 5’-TGATATATAAGGCCCGGACA-3’ |
| **NPM1/B23** | 5’-AAGGTGAATCGAGGTGCTCT-3’ |
|  | 5’-CTGTGTAAGATATGGCGGGTT-3’ |
| **β-actin** | 5’-AGAAAATCTGGCACCACACC-3’ |
|  | 5’-CCATCTCTTGCTCGAAGTCC-3’ |

**Supplementary Table 10**. Two-by-two contingency table for gene pairs of a particular network and those of other networks in a function gene set.

|  | **Gene pairs of a network** | **Gene pairs of other networks** | **Total** |
| --- | --- | --- | --- |
| **In gene set** | *k* | *K-k* | *K* |
| **Not in gene set** | *h-k* | *(H-K)-(h-k)* | *H-K* |
| **Total** | *h* | *H-h* | *H* |

Supplementary Methods and Data

Independent simulations were performed to verify that adopting a critical value *D_α_* for D and a fixed coefficient γ maintains good control of type I error rate for any number M of genes. More specifically, we defined the critical value of D as:


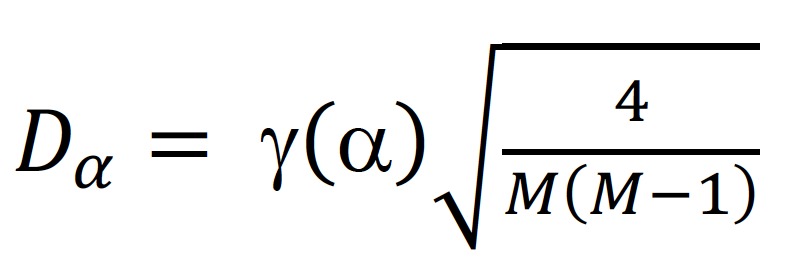


We considered M = 10, 20, …, 100, 200 and 500 in the simulations. In each experiment, 1000 M × M correlation matrices were generated following the algorithm proposed by Numpacharoen and Atsawarungruangkit (2012)^40^. This algorithm uses any bounded random variable to generate positive semi-definite correlation matrices with a flexible distribution of entries. It was found that using a beta distribution with parameters a=b=2 produced a distribution of correlation coefficients similar to that of the observed data. Therefore, we first generated correlation matrices under this setting. Applying the Cholesky decomposition, each correlation matrix was then used to randomly generate two independent matrices, which represent the gene expression matrices for normal and neoplastic state. Finally, two-sample KS test were performed to obtain the distribution of D under the null hypothesis. The critical value *D*_0.05_ was identified as the smallest D value such that the empirical type I error was less than 0.05. For each M, the value of γ was estimated according to the following formula.


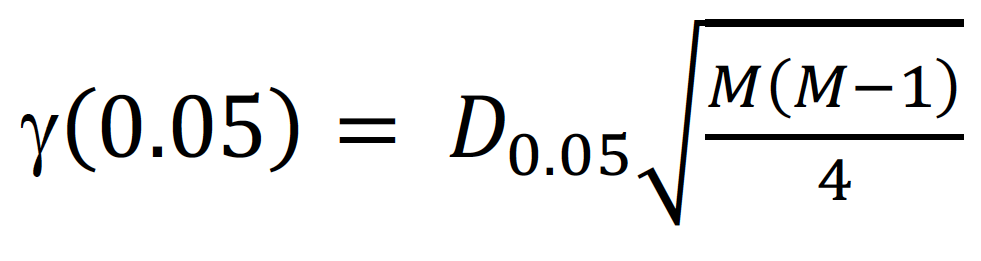


The plot of coefficient γ against gene number M is shown in Supplementary Fig. 7. It was found that the value of γ increases as a function of M, but plateaus around 3.1.

We also performed an independent set of simulations to estimate and compare the type I and type II error rates for M = 100 and γ = 1.36, 3.1. For type I error simulations, we continued to use a beta distribution with parameters a=b=2 to produce correlation matrices for both normal and neoplastic states. For type II error simulations, we used a beta distribution with parameters a=b=1 to produce correlation matrices for neoplastic states. Type I and type II error rates for γ = 1.36 were found to be 0.488 and 0.031, while that for γ = 3.1 were 0.048 and 0.231. Our simulation results suggest that type I error rate is controlled below 0.05 and that the statistical power, though reduced, is retained above 0.77 by adopting the corrected coefficient γ = 3.1.
